# Gene-based analysis in HRC imputed genome wide association data identifies three novel genes for Alzheimer’s disease

**DOI:** 10.1371/journal.pone.0218111

**Published:** 2019-07-08

**Authors:** Emily Baker, Rebecca Sims, Ganna Leonenko, Aura Frizzati, Janet C. Harwood, Detelina Grozeva, GERAD/PERADES, CHARGE, ADGC, EADI, IGAP consortia, Kevin Morgan, Peter Passmore, Clive Holmes, John Powell, Carol Brayne, Michael Gill, Simon Mead, Paola Bossù, Gianfranco Spalletta, Alison M. Goate, Carlos Cruchaga, Wolfgang Maier, Reinhard Heun, Frank Jessen, Oliver Peters, Martin Dichgans, Lutz FröLich, Alfredo Ramirez, Lesley Jones, John Hardy, Dobril Ivanov, Matthew Hill, Peter Holmans, Nicholas D. Allen, B. Paul Morgan, Sudha Seshadri, Gerard D. Schellenberg, Philippe Amouyel, Julie Williams, Valentina Escott-Price

**Affiliations:** 1 Medical Research Council Centre for Neuropsychiatric Genetics and Genomics, Division of Psychological Medicine and Clinical Neurosciences, Cardiff University, Cardiff, United Kingdom; 2 UK Dementia Research Institute at Cardiff University, Cardiff, United Kingdom; 3 Human Genetics, School of Life Sciences, Life Sciences Building A27, University Park, University of Nottingham, Nottingham, NG7 2RD, United Kingdom; 4 Centre for Public Health, School of Medicine, Dentistry and Biomedical Sciences, Queens University, Belfast, United Kingdom; 5 Division of Clinical Neurosciences, School of Medicine, University of Southampton, Southampton, United Kingdom; 6 Department of Basic and Clinical Neuroscience, Institute of Psychiatry, Psychology and Neuroscience, Kings College London, London, United Kingdom; 7 Genetic Epidemiology, QIMR Berghofer Medical Research Institute, Herston, Queensland, Australia; 8 Institute of Public Health, University of Cambridge, Cambridge, United Kingdom; 9 Mercer’s Institute for Research on Ageing, St. James’ Hospital, Dublin, Ireland; 10 James Hospital and Trinity College, Dublin, Ireland; 11 MRC Prion Unit at UCL, Institute of Prion Diseases, London, United Kingdom; 12 Experimental Neuropsychobiology Laboratory, IRCCS Santa Lucia Foundation, Department of Clinical and Behavioral Neurology, Rome, Italy; 13 Laboratory of Neuropsychiatry, IRCCS Santa Lucia Foundation, Rome, Italy; 14 Icahn School of Medicine at Mount Sinai, New York, New York, United States of America; 15 Hope Center Program on Protein Aggregation and Neurodegeneration, Washington University School of Medicine, St Louis, Missouri, United States of America; 16 Department of Psychiatry, Washington University School of Medicine, St Louis, Missouri, United States of America; 17 German Centre for Neurodegenerative Diseases (DZNE), 53127 Bonn, Germany; 18 Department of Psychiatry and Psychotherapy, University of Bonn, 53127, Bonn, Germany; 19 Department of Psychiatry and Psychotherapy, University of Cologne, 50937 Cologne, Germany; 20 Department of Psychiatry and Psychotherapy, Charité Berlin, Berlin, Germany; 21 German Center for Neurodegenerative Diseases (DZNE), Berlin, Germany; 22 Institute for Stroke and Dementia Research, Klinikum der Universität München, Munich, Germany; 23 German Center for Neurodegenerative Diseases (DZNE, Munich), Munich, 80336, Germany; 24 Munich Cluster for Systems Neurology (SyNergy), Munich, Germany; 25 Central Institute of Mental Health, Medical Faculty Mannheim, University of Heidelberg, Heidelberg, Germany; 26 Department for Neurodegenerative Diseases and Geriatric Psychiatry, University Hospital Bonn, Bonn, Germany; 27 Department of Molecular Neuroscience, UCL, Institute of Neurology, London, United Kingdom; 28 Department of Neurology, Boston University School of Medicine, Boston, Massachusetts, United States of America; 29 Department of Pathology and Laboratory Medicine, University of Pennsylvania Perelman School of Medicine, Philadelphia, Pennsylvania, United States of America; 30 Univ. Lille, Inserm, CHU Lille University Hospital, Institut Pasteur de Lille, LabEx DISTALZ-UMR1167 - RID-AGE - Risk factors and molecular determinants of aging-related, F-59000 Lille, France; University of Ioannina Medical School, GREECE

## Abstract

Late onset Alzheimer’s disease is the most common form of dementia for which about 30 susceptibility loci have been reported. The aim of the current study is to identify novel genes associated with Alzheimer’s disease using the largest up-to-date reference single nucleotide polymorphism (SNP) panel, the most accurate imputation software and a novel gene-based analysis approach which tests for patterns of association within genes, in the powerful genome-wide association dataset of the International Genomics of Alzheimer’s Project Consortium, comprising over 7 million genotypes from 17,008 Alzheimer’s cases and 37,154 controls. In addition to earlier reported genes, we detected three novel gene-wide significant loci *PPARGC1A* (*p* = 2.2 × 10^−6^), *RORA* (*p* = 7.4 × 10^−7^) and *ZNF423* (*p* = 2.1 × 10^−6^). *PPARGC1A* and *RORA* are involved in circadian rhythm; circadian disturbances are one of the earliest symptoms of Alzheimer’s disease. *PPARGC1A* is additionally linked to energy metabolism and the generation of amyloid beta plaques. *RORA* is involved in a variety of functions apart from circadian rhythm, such as cholesterol metabolism and inflammation. The *ZNF423* gene resides in an Alzheimer’s disease-specific protein network and is likely involved with centrosomes and DNA damage repair.

## Introduction

Late Onset Alzheimer’s disease (LOAD) is a devastating neurodegenerative condition with significant genetic heritability [1]. The apolipoprotein E (*APOE*) gene is the strongest genetic risk factor for LOAD [2]. Subsequently, more genes were found to be associated with AD development. The Genetic and Environmental Risk in Alzheimer’s Disease (GERAD) Consortium published a Genome-Wide Association Study (GWAS) that identified novel variants in *CLU* and *PICALM* which were associated with AD [3]. Concurrently, the European Alzheimer’s Disease Initiative (EADI) identified an association between the *CR1* and *CLU* loci and AD [4]. Subsequent publications by GERAD, the Alzheimer’s Disease Genetic Consortium (ADGC) and Cohorts for Heart and Aging Research in Genomic Epidemiology (CHARGE) Consortium identified a further 5 novel loci [5] [6] [7]. The International Genomics of Alzheimer’s Project (IGAP) [4] Consortium is an amalgamation of these four different genetic groups (GERAD, EADI, ADGC and CHARGE). Meta-analysis of the 4 GWAS datasets determined 11 novel variants associated with AD. A gene-based analysis has been undertaken in the IGAP AD data using Brown’s method [8]. This approach determined two additional novel genes; *TP53INP1* and *IGHV1-67* [9]. Additionally, low frequency risk variants have been identified through next generation sequencing (*TREM2*) [10] and a whole-exome association study (*PLCG2*, *TREM2* and *ABI3* [11]).

Gene-based analysis is an alternative to GWAS analyses, which considers the association of an individual single nucleotide polymorphism (SNP) with disease. Gene-based analyses provide more power due to the aggregate effect of multiple SNPs being larger than that of individual SNPs. For example, determining the association of genes rather than SNPs, is beneficial since genes are more robust across different populations, this is due to the linkage disequilibrium (LD) between SNPs resulting in different SNPs being associated in different populations [12]. Gene-based analyses are being widely used in the field and as expected, are able to identify novel genes or pathways associated with disease. Pathways clustering in eight areas of biology have been found to be associated with AD using the ALIGATOR [13] algorithm [14] [15].

The aim of the current study is to identify novel genes associated with AD using the largest up-to-date reference SNP panel, the most accurate imputation software and a novel gene-based analysis approach. In this study, we used the GERAD data [3] which have been imputed using the latest Haplotype Reference Consortium data (HRC). Polygenic Linkage disequilibrium- Adjusted Risk Score (POLARIS) [16] is a powerful gene-based method which produces a risk score per person per gene, adjusts for LD between SNPs and informs the analysis with summary statistics from an external data set. POLARIS, unlike standard Polygenic Risk Score (PRS) does not require data to be pruned for LD prior to analysis, so it is able to incorporate information from a larger number of SNPs. We employed the POLARIS approach [16] and using the individual genotypes in the GERAD imputed data, produced the risk score for each individual for every gene considered. The IGAP [4] SNP summary statistic data, where the individuals from GERAD were excluded, were used to generate the gene-based PRS.

## Results

**Fig 1:**
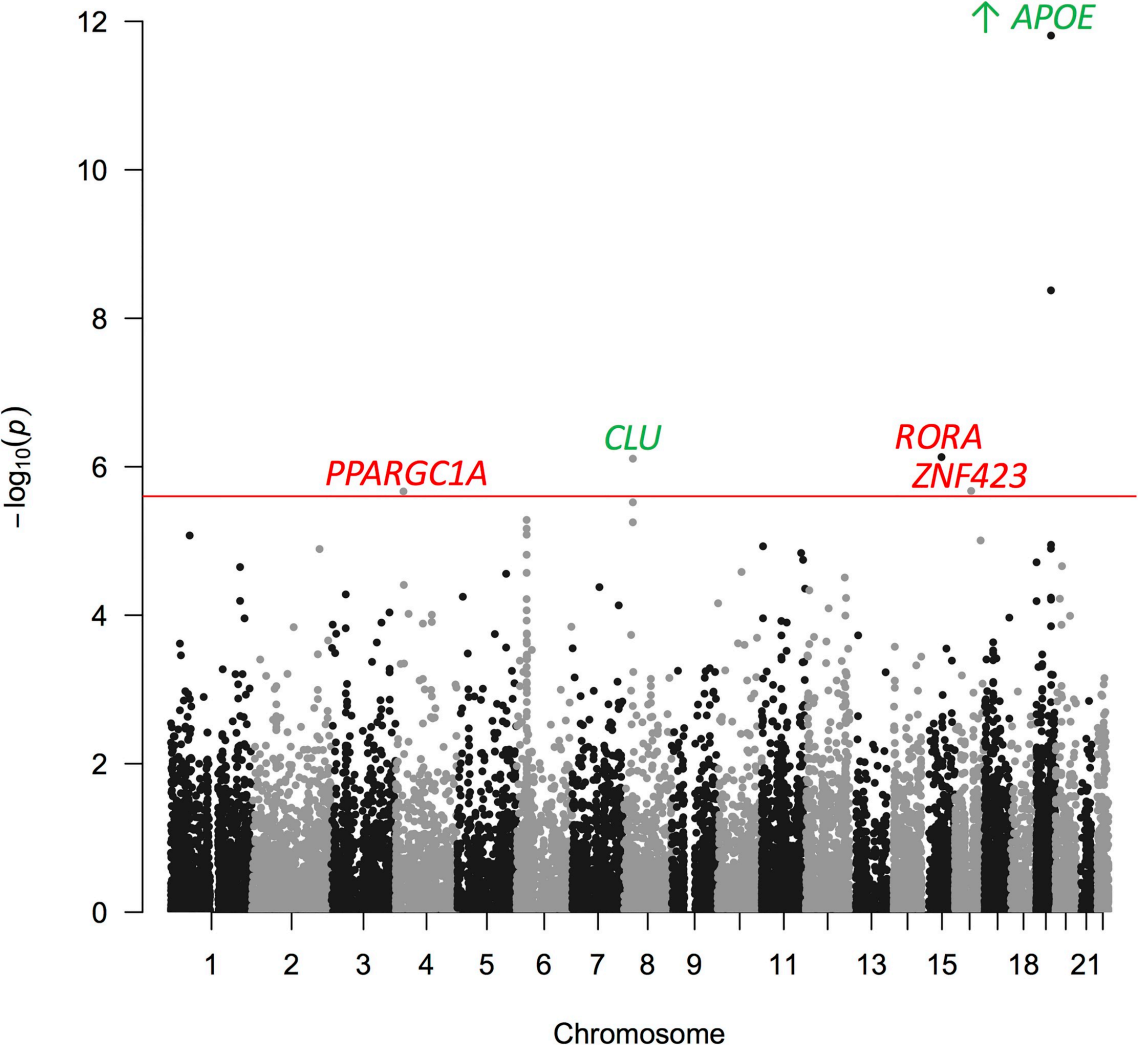
Manhattan Plot for the POLARIS Gene-Based Analysis in Imputed GERAD Data Using a Gene Window 35kb Upstream and 10kb Downstream.

For the imputed GERAD data, using a window around the gene of 35kb upstream and 10kb downstream [17], SNPs are assigned to 18,087 genes which are plotted on a Manhattan plot in Fig 1. The 12 gene-wide significant genes from this analysis are shown in Table 1, where gene-wide significance is defined as *p* < 2.5 × 10^−6^ [18]. A large number of genes reside on chromosome 19 and these are likely influenced by the large effect of *APOE*. Three novel genes have been identified from this analysis: *PPARGC1A*, *RORA* and *ZNF423*. *PPARGC1A* (peroxisome proliferator-activated receptor gamma co-activator 1alpha) is a master regulator that mainly regulates energy metabolism [19] [20]. It has been linked to the generation of amyloid beta plaques [21] and circadian rhythm [22]. *RORA* (Retinoic acid receptor-related orphan receptor alpha) is involved in a variety of functions such as circadian rhythm, cholesterol metabolism and inflammation [23]. Its expression is also upregulated in the AD hippocampus [24]. The *ZNF423* gene resides in an AD-specific protein network which also includes other AD-related genes such as *APOE*, *CLU*, *ABCA7*, *TREM2* etc. [25]. *ZNF423* is likely involved with centrosomes and DNA damage repair [26]. The *SCARA3* gene overlaps *CLU* which has previously been identified as being associated with AD [3] [4]. The POLARIS gene-based results for the genes previously identified as being associated with AD are seen in S1 Table, these genes contain genome-wide significant SNPs (*p* < 5 × 10^−8^). Table 1 additionally shows the POLARIS gene-based results conditioned on the *APOE* gene, this is done by including the POLARIS *APOE* gene risk score into the logistic regression model. *ZNF423* conditioned on *APOE* no longer reaches gene-wide significance, but *PPARGC1A* and *RORA* remain significant, suggesting an association independent of *APOE*. In addition, *BCAM*, *PVRL2* and *APOC4-APOC2* on chromosome 19 remain gene-wide significant, even after adjusting for *APOE* in the model, suggesting a potential signal beyond *APOE*. To investigate this, we additionally conditioned on *BCAM* (the most significant chromosome 19 gene after adjusting for *APOE*) to determine whether this explains the remaining effect. Results are shown in S2 Table; when conditioning on *APOE* and *BCAM* the only remaining gene-wide significant gene on chromosome 19 is *APOC4-APOC2*, suggesting that the majority of signals on chromosome 19 are explained by *APOE* and *BCAM*. We were unable to condition on *APOE* genotype since these are not available for all subjects, so removal of an association may be due to reduced sample size.

**Table 1:** Gene-Wide Significant Genes from POLARIS Gene-based Analysis in GERAD Imputed Data Using a Gene Window (35kb Upstream and 10kb Downstream).

			POLARIS			POLARIS, conditioned on *APOE*		
Chr	Gene	No. of SNPs	Beta	SE	P-value	Beta	SE	P-value
4	*PPARGC1A*	480	0.877	0.1851	2.2 × 10^−6^	0.920	0.1885	1.0 × 10^−6^
8	*SCARA3* (*CLU*)	240	0.526	0.1064	7.8 × 10^−7^	0.537	0.1090	8.3 × 10^−7^
15	*RORA*	1813	0.334	0.0674	7.4 × 10^−7^	0.338	0.0688	9.1 × 10^−7^
16	*ZNF423*	1056	0.551	0.1163	2.1 × 10^−6^	0.541	0.1187	5.1 × 10^−6^
19	*BCL3*	88	0.377	0.0674	4.2 × 10^−9^	0.291	0.0656	8.8 × 10^−6^
19	*CBLC*	50	0.605	0.1161	1.8 × 10^−7^	0.455	0.1183	0.00012
19	*BCAM*	71	0.556	0.0543	1.4 × 10^−24^	0.492	0.0555	7.6 × 10^−19^
19	*PVRL2*	160	0.546	0.0299	9.4 × 10^−75^	0.430	0.0491	2.0 × 10^−18^
19	*TOMM40*	108	0.500	0.0298	3.4 × 10^−63^	0.334	0.0891	0.00018
19	*APOE*	55	0.520	0.0315	4.4 × 10^−61^	NA	NA	NA
19	*APOC1*	34	0.475	0.0315	1.5 × 10^−51^	-0.249	0.1031	0.01575
19	*APOC4-APOC2*	62	0.615	0.0871	1.6 × 10^−12^	0.419	0.0892	2.5 × 10^−6^

In order to narrow down the disease-associated SNPs for each of these novel genes, we investigated the gene expression patterns using the BRAINEAC [27] database from the UK Brain Expression Consortium. For the *PPARGC1A* gene the SNP rs67436520, which is downstream of the *PPARGC1A* gene, has the best cis-expression quantitative trait loci (eQTL) p-value of 3.3 × 10^−4^, this is expressed in the hippocampus. The best cis-eQTL p-value in the *RORA* gene is 1.5 × 10^−4^, this is for SNP rs113223478 which is 78.5kb upstream of the gene, between the *NARG2* and *ANXA2* genes, and is expressed in the substantia nigra. This SNP will not be included in the POLARIS score, however, it could be tagged by SNPs included in the score. Finally, SNP rs2270396 has the best cis-eQTL p-value in the *ZNF423* gene with a p-value of 3.0 × 10^−5^ and is expressed in the frontal cortex. These SNPs were checked in RegulomeDB [28] and Variant Effect Predictor [29]. They are all intergenic variants that are not in any well-defined regulatory region of the genome and do not overlap the best risk SNPs, so it is difficult to predict how these SNPs may affect the regulation of the expression of these genes.

## Discussion

A gene-based analysis was performed using the individual genotypes in the GERAD imputed data and the summary statistics from IGAP data excluding the GERAD subjects was used to inform the analysis. This analysis expands a gene window around the gene, 35kb upstream and 10kb downstream, which is likely to include transcriptional regulatory elements in the gene [17] and thus contain SNPs influencing gene expression. Three novel genes were found to be associated with AD using the POLARIS method. The novel genes are *PPARGC1A*, *RORA* and *ZNF423*, all of which have credible biological relevance to AD. These results are already adjusted for LD between SNPs in the gene, using the POLARIS methodology. Most of the genes identified before in IGAP data [4] [9] were also identified by POLARIS as statistically significant, however, since previous results are based on IGAP stages 1 and 2, POLARIS p-values were slightly larger.

We investigated disease-associated SNPs using expression patterns, which highlighted individual SNPs. A limitation of this analysis is that the POLARIS score tests the aggregated risk across the gene and is unlikely due to a single SNP.

The product of the *PPARGC1A* gene, PGC-1*α* (Peroxisome proliferator-activated receptor gamma coactivator 1-alpha) is part of the PGC-1 family of transcriptional coactivators that mainly regulate mitochondrial biogenesis to in turn regulate the cellular energy metabolism [19]. It is also involved in other cellular and physiological functions, including the response to a variety of cellular and external stimuli, cellular glucose homeostasis, circadian rhythm, and the regulation of neuronal apoptosis.

The regulation of this gene is complex; it has multiple isoforms and alternative promoters [30] and gene expression is regulated by a variety of stimuli, including cytokines, insulin, exercise and the cold [31]. PGC-1*α* can induce ribosomal transcription under stress conditions such as oxidative stress and exercise [32].

Previous animal model work has shown that overexpression of hPGC-1*α* in APP23 mice improved spatial and recognition memory, along with a significant reduction of A*β* deposition [21]. Furthermore, hPGC-1*α* overexpression also reduced the levels of proinflammatory cytokines and microglial activation [21] [33]. This suggests a direct link with recent genetic evidence of microglia-mediated innate immune response involvement in AD [11]. In addition, activation of PGC-1*α* by EKR and p38 inhibitors have been shown to improve spatial and learning memory in A*β*-injected rats [34]. *PPARGC1A* has also been implicated in the pathogenesis of other neurodegenerative disorders, namely Huntington’s and Parkinson’s diseases [35]. It has been shown that mutated Huntingtin represses PGC-1*α*, affecting mitochondrial function, hence ribosomal biogenesis may be affected in Huntington’s disease [36]. There is a brain specific promoter 587kb upstream of human *PPARGC1A* [37], which is located in a genomic region associated with age of onset of Huntington’s disease and relevant here is that hippocampal PGC-1*α* expression is decreased in the AD brain [38]. A randomised controlled trial of a PPAR-*γ* agonist, pioglitazone, found improved cognition and regional cerebral blood flow in patients with mild AD [39].

*RORA* (Retinoic acid receptor-related orphan receptor alpha) is a nuclear hormone receptor with diverse cellular roles [40], for example in immunity, cerebellum development [41], lipid metabolism [42], circadian rhythms and inflammation [23]. *RORA* regulates its target genes by binding to the ROR response elements (RORE) in the gene regulatory region [43]. It has been shown to regulate more than 3,000 genes in human monocytic and endothelial cell lines [44]. It has a role in the regulation of the BDNF pathway and its expression is upregulated in AD hippocampus [24]. *RORA* and *PPARGC1A* are close biological partners, with PGC-1*α* regulating the expression of a number of clock genes through the coactivation of the ROR family of orphan nuclear receptors [45]. *RORA* has been shown to be linked to other genes previously implicated in AD [25] and also has been implicated in a large number of neuropsychiatric disorders, such as post-traumatic stress disorder [46] [47] and autism [48]. Furthermore, *RORA* trans-activates IL-6 and is thought to be neuro-protective in astrocytes and anti-inflammatory in peripheral tissues [49]. The two genes, *RORA* and *PPARGC1A* that we report here provide further evidence of the involvement of inflammation in the pathogenesis of AD.

Finally, *ZNF423* is a nuclear protein that belongs to the Kruppel-like C2H2 zinc finger proteins. *ZNF423* directs bone morphogenetic protein (BMP)-dependent signalling activity and aberrant forms impede B cell differentiation [50]. Furthermore, elevated gene-expression of *ZNF423* has been shown to occur in patients with systemic lupus erythematosus, pointing to an impaired function of B cells in human mesenchymal stem cells [51]. *ZNF423* resides in an AD-specific protein network [25]. *ZNF423* is likely involved with centrosomes and DNA damage repair [26]. It is downregulated in human neuroblastoma and glioma [52] [53] and also has a role in breast cancer [54]. Previously, it also has been shown that missense and LoF variants are likely to be pathogenic for abnormality of brain morphology, Joubert syndrome and Nephronophthisis with autosomal dominant or autosomal recessive inheritance (www.omim.org, https://www.ncbi.nlm.nih.gov/clinvar/). These disorders present with a range of phenotypic characteristics, with the central nervous system being affected too (more specifically the cerebellar vermis). In nur12 mouse model (with introduced nonsense mutation in exon 4 of the mouse Zfp423 gene), Alcaraz et al. [55] observed loss of the corpus callosum, reduction of hippocampus, and a malformation of the cerebellum reminiscent of patients with Dandy-Walker syndrome. Within the cerebellum, Zfp423 was observed to be expressed in both ventricular and external germinal zones. Loss of Zfp423 was also observed to lead to diminished proliferation by granule cell precursors in the external germinal layer and abnormal differentiation and migration of ventricular zone-derived neurons and Bergmann glia [55].

## Conclusion

POLARIS is a gene-based analysis which produces a genetic risk score per individual per gene, whilst adjusting for LD between SNPs in the gene. This methodology was applied to the latest HRC imputation of the GERAD data, and the summary statistics from IGAP (excluding GERAD subjects) were used as weights in the score. This led to the identification of 3 novel genes associated with AD; these genes are *PPARGC1A*, *RORA* and *ZNF423*. There is evidence that these genes are credible candidates in AD, with *PPARGC1A* and *RORA* being linked to circadian rhythm, *PPARGC1A* is implicated in energy metabolism and the generation of amyloid plaques, *RORA* is linked to cholesterol metabolism and inflammation and *ZNF423* is likely involved in DNA damage repair and resides in an AD-specific protein network.

## Materials and methods

The Haplotype Reference Consortium (HRC), version r1.1 2016, was used to impute GERAD genotype data on the Michigan Imputation Server [56], which to date, allows the most accurate imputation of genetic variants. Imputed genotype probabilities (also known as dosages) were converted to the most probable genotype with a probability threshold of 0.9 or greater. SNPs were removed if: their imputation INFO-score< 0.4, minor allele frequency (MAF)< 0.01, missingness of genotypes≥ 0.05 or HWE< 10^−6^. A total of 6,119,694 variants were retained. To correct for population structure and genotyping differences, all analyses were adjusted for age, gender and the top 3 principal components.

POLARIS was applied to this GERAD (3,332 cases, 9,832 controls; see S3 Table for cohort details) imputed data, using the IGAP [4] data (17,008 cases, 37,154 controls) excluding GERAD subjects (IGAPnoGERAD) as an external dataset to derive weights from the best powered data set avaliable. The IGAP data was imputed using a previous reference panel (1000 genomes, Dec 2010 release). There were 3,169,839 SNPs in common between imputed GERAD and IGAP summary statistics data. The GERAD imputed data contain individual genotypes for every SNP, enabling the production of a risk score per person per gene, and the IGAPnoGERAD data contains effect sizes for every SNP, which are used to weight the risk score. A gene-based risk score was produced for every individual in the GERAD data.

POLARIS adjusts for LD between SNPs and therefore, the SNPs were not pruned for LD and the entire data were used in this analysis. POLARIS adjusts for LD by using spectral decomposition of the correlation matrix between SNPs. Such a matrix was derived for each gene using the individual genotypes from the GERAD imputed data. It was ensured that SNPs had consistent reference alleles across both independent datasets; IGAPnoGERAD and imputed GERAD. If alleles in IGAPnoGERAD were coded in the opposite direction to those in GERAD, the summary effect size for the SNP was inverted. SNPs with alleles AT, TA, CG or GC were excluded.

SNPs were assigned to genes using GENCODE (v19) gene models [57]. Only genes with known gene status and those marked as protein coding were used. A gene window containing SNPs which were within 35kb upstream and 10kb downstream of the gene was considered. This window was used since it is likely to contain transcriptional regulatory elements [17]. SNPs which belong to multiple genes were assigned to all those genes. In the HRC imputed GERAD data, 2,296,690 SNPs were assigned to 18,087 genes.

A POLARIS score was produced for each of these genes, and the overall association of the gene with AD is determined using a logistic regression model, adjusting for population covariates, age and sex.

## Supporting information

S1 Appendix Authors who contributed to the generation of original study data for GERAD, ADGC, CHARGE and EADI. (DOCX)

S1 Table POLARIS Gene-Based Results for GWAS Associated Genes. (PDF)

S2 Table Gene-Wide Significant Genes from POLARIS Gene-based Analysis Conditioned on *APOE* and *BCAM* and best IGAP SNP and p-value. (PDF)

S3 Table GERAD Cohort Descriptives and Sample Size. (PDF)
