# Supplementary material for: Gene-based analysis in HRC imputed genome wide association data identifies three novel genes for Alzheimer’s disease
Source: PLoS One. 2019 Jul 8;14(7):e0218111. doi: 10.1371/journal.pone.0218111 (PMC6613773; doi:10.1371/journal.pone.0218111)
Supplement: S1 Appendix — (DOCX) [file pone.0218111.s001.docx]

**S1 Appendix. Authors who contributed to the generation of original study data for GERAD, ADGC, CHARGE and EADI.**

**Genetic and Environmental Risk in AD/Defining Genetic, Polygenic and Environmental Risk for Alzheimer's Disease Consortium (GERAD/PERADES) Consortium**

Rebecca Sims, Maria Vronskaya, Aura Frizatti, Nandini Badarinarayan, Rachel Raybould, Taniesha Morgan, Per Hoffmann, Denise Harold, Amy Gerrish, Nicola Denning, Nick C. Fox, Joseph T. Hughes, Yogen Patel, Makrina Daniilidou, James Uphill, Daniela Galimberti, Elio Scarpini, Johannes Kornhuber, Sabrina Sordon, Manuel Mayhaus, Wei Gu, Annette M Hartmann, Simon Lovestone, Rebecca Sussams, Clive Holmes, Wolfgang Maier, Amit Kawalia, Susanne Moebus, James Turton, Jenny Lord, Iwona Kloszewska, Aoibhinn Lynch, Brian Lawlor, Michael Gill, Monica Diez-Fairen, Ignacio Alvarez, Antonio Ciaramella, Chiara Cupidi, Raffaele Giovanni Maletta, Roberta Cecchetti, Magda Tsolaki, David Craig, Despoina Avramidou, Antonia Germanou, Maria Koutroumani, Olymbia Gkatzima, Harald Hampel, David C. Rubinsztein, Lutz Frölich, Bernadette McGuinness, Janet A. Johnston, Peter Passmore, Jonathan M. Schott, Jason D. Warren, Michelle K. Lupton, Petra Proitsi, John Powell, John S. K. Kauwe, Michelangelo Mancuso, Ubaldo Bonuccelli, Andrew McQuillin, Gill Livingston, Nicholas J. Bass, John Hardy, Jose Bras, Minerva M. Carrasquillo, Rita Guerreiro, Elizabeth Fisher, Carlo Masullo, Gina Bisceglio, Li Ma, Neill R. Graff-Radford, Angela Hodges, Martin Scherer, Matthias Riemenschneider, Reinhard Heun, Heike Kölsch, Markus Leber, Isabella Heuser, Ina Giegling, Michael Hüll, John Morris, Kevin Mayo, Thomas Feulner, Dmitriy Drichel, Thomas D. Cushion, Paul Hollingworth, Rachel Marshall, Alun Meggy, Georgina Menzies, Ganna Leonenko, Detelina Grozeva, Giancarlo Russo, Frank Jessen, Bruno Vellas, Emma Vardy, Karl-Heinz Jöckel, Martin Dichgans, David Mann, Stuart Pickering-Brown, Norman Klopp, H-Erich Wichmann, Kevin Morgan, Kristelle Brown, Christopher Medway, Markus M. Nöthen, Nigel M. Hooper, Antonio Daniele, Anthony Bayer, John Gallacher, Hendrik van den Bussche, Carol Brayne, Steffi Riedel-Heller, Ammar Al-Chalabi, Christopher E. Shaw, Jens Wiltfang, Victoria Alvarez, Andrew B. Singleton, John Collinge, Simon Mead, Martin Rossor, Natalie S. Ryan, Benedetta Nacmias, Sandro Sorbi, Eleonora Sacchinelli, Gianfranco Spalletta, Carlo Caltagirone, Maria Donata Orfei, Robert Clarke, A. David Smith, Donald Warden, Gordon Wilcock, Amalia Cecilia Bruni, Maura Gallo, Yoav Ben-Shlomo, Patrizia Mecocci, Pau Pastor, Oliver Peters, Virginia Boccardi, Nick Warner, Panagiotis Deloukas, Carlos Cruchaga, Rhian Gwilliam, Patrick G. Kehoe, Seth Love, Chris Corcoran, JoAnn Tschanz, Ron Munger, Michael C. O’Donovan, Lesley Jones, Michael J. Owen, Valentina Escott-Price, Alfredo Ramirez, Peter A. Holmans, Julie Williams

**Alzheimer Disease Genetics Consortium (ADGC) Consortium**

Brian W. Kunkle, Adam C. Naj, Alexandre Amlie-Wolf, Eden R. Martin, Kara L. Hamilton-Nelson, Amanda B. Kuzma, Badri N. Vardarajan, Gary W. Beecham, Otto Valladares, Liming Qu, Yi Zhao, John Malamon, Beth A. Dombroski, Shubhabrata Mukherjee, Laura B. Cantwell, Mariusz Butkiewicz, Will Bush, Yeunjoo Song, Robert C. Barber, Perrie M. Adams, Marilyn S. Albert, Duane Beekly, Deborah Blacker, Rachelle S. Doody, Thomas J. Fairchild, Matthew P. Frosch, Bernardino Ghetti, Ryan M. Huebinger, M. Ilyas Kamboh, Mindy J. Katz, C. Dirk Keene, Walter A. Kukull, Eric B. Larson, Richard B. Lipton, Thomas J. Montine, Ronald C. Petersen, Eric M. Reiman, Joan S. Reisch, Donald R. Royall, Mary Sano, Peter St George-Hyslop, Debby W. Tsuang, Chuang-Kuo Wu, Roger L. Albin, Liana G. Apostolova, Steven E. Arnold, Sanjay Asthana, Patrice Whitehead, Craig S. Atwood, Lisa L. Barnes, Sandra Barral, Thomas G. Beach, James T. Becker, Eileen H. Bigio, Thomas D. Bird, Bradley F. Boeve, James D. Bowen, Adam Boxer, James R. Burke, Jeffrey M. Burns, Joseph D. Buxbaum, Nigel J. Cairns, Chuanhai Cao, Chris S. Carlson, Cynthia M. Carlsson, Regina M. Carney, Minerva M. Carrasquillo, Mariet Allen, Helena C. Chui, David H. Cribbs, Elizabeth A. Crocco, Charles DeCarli, Malcolm Dick, Ranjan Duara, Neill R Graff-Radford, Denis A. Evans, Kelley M. Faber, Kenneth B. Fallon, David W. Fardo, Martin R. Farlow, Steven Ferris, Tatiana M. Foroud, Douglas R. Galasko, Marla Gearing, Daniel H. Geschwind, John R. Gilbert, Robert C. Green, John H. Growdon, Ronald L. Hamilton, Lindy E. Harrell, Lawrence S. Honig, Matthew J. Huentelman, Christine M. Hulette, Bradley T. Hyman, Gail P. Jarvik, Erin Abner, Lee-Way Jin, Gyungah R. Jun, Clinton T. Baldwin, Anna Karydas, Jeffrey A. Kaye, Ronald Kim, Neil W. Kowall, Joel H. Kramer, Frank M. LaFerla, James J. Lah, James B. Leverenz, Allan I. Levey, Andrew P. Lieberman, Kathryn L. Lunetta, Constantine G. Lyketsos, Daniel C. Marson, Frank Martiniuk, Deborah C. Mash, Eliezer Masliah, Wayne C. McCormick, Susan M. McCurry, Andrew N. McDavid, Ann C. McKee, Marsel Mesulam, Bruce L. Miller, Carol A. Miller, Joshua W. Miller, John C. Morris, Amanda J. Myers, Sid O’Bryant, John M. Olichney, Joseph E. Parisi, Henry L. Paulson, William R. Perry, Elaine Peskind, Aimee Pierce, Wayne W. Poon, Huntington Potter, Joseph F. Quinn, Ashok Raj, Murray Raskind, Barry Reisberg, Christiane Reitz, John M. Ringman, Erik D. Roberson, Ekaterina Rogaeva, Howard J. Rosen, Roger N. Rosenberg, Mark A. Sager, Andrew J. Saykin, Michael L. Cuccaro, Jeffery Vance, Julie A. Schneider, Lon S. Schneider, Susan Slifer, William W. Seeley, Amanda G. Smith, Joshua A. Sonnen, Salvatore Spina, Robert A. Stern, Russell H. Swerdlow, Mitchell Tang, Rudolph E. Tanzi, John Q. Trojanowski, Juan C. Troncoso, Vivianna M. Van Deerlin, Linda J. Van Eldik, Harry V. Vinters, Jean Paul Vonsattel, Sandra Weintraub, Kathleen A. Welsh-Bohmer, Kirk C. Wilhelmsen, Jennifer Williamson, Thomas S. Wingo, Randall L. Woltjer, Clinton B. Wright, Chang-En Yu, Lei Yu, Paul K. Crane, David Bennett, Philip L. De Jager, Carlos Cruchaga, Alison M. Goate, Nilufer Ertekin-Taner, Steven G. Younkin, Dennis W. Dickson, Hakon Hakonarson, Lindsay A. Farrer, Jonathan Haines, Richard Mayeux, Li-San Wang, Gerard D. Schellenberg, Margaret A. Pericak-Vance

**The European Alzheimer's Disease Initiative (EADI) Consortium**

Benjamin Grenier-Boley, Vincent Damotte, Anne Boland, Céline Bellenguez, Kristel Sleegers, Robert Olaso, Mikko Hiltunen, Jacques Epelbaum, Jean-Guillaume Garnier, Marie-Laure Moutet, Delphine Bacq, Maria Del Zompo, Ignacio Mateo, Florentino Sanchez-Garcia, Maria Candida Deniz Naranjo, David Wallon, Fabienne Garzia, Bertrand Fin, Stéphane Meslage, Sebastiaan Engelborghs, Rik Vandenberghe, Peter De Deyn, Alessio Squassina, Eloy Rodriguez-Rodriguez, Carmen Munoz-Fernadez, Yolanda Aladro Benito, Hakan Thonberg, Vilmantas Giedraitis, Lena Kilander, RoseMarie Brundin, Letizia Concari, Seppo Helisalmi, Anne Maria Koivisto, Annakaisa Haapasalo, Vincenzo Solfrizzi, Vincenza Frisardi, Vincent Deramecourt, Francesca Salani, Nathalie Fievet, Olivier Hanon, Carole Dufouil, Alexis Brice, Karen Ritchie, Bruno Dubois, Hilkka Soininen, Laura Fratiglioni, Lina Keller, Francesco Panza, Didier Hannequin, Paolo Caffarra, Lars Lannfelt, Florence Pasquier, Paola Bossù, Alberto Pilotto, Maria J. Bullido, Paola Bosco, Eliecer Coto, Alberto Lleo, Martin Ingelsson, Caroline Graff, Pascual SanchezJuan, Claudine Berr, Stéphanie Debette, Jean-Francois Dartigues, Gael Nicolas, Dominique Campion, Jordi Clarimon, Christine Van Broeckhoven, Jean-François Deleuze, Phillippe Amouyel, Jean-Charles Lambert

**Cohorts for Heart and Aging Research in Genomic Epidemiology Consortium (CHARGE) Consortium**

Joshua C. Bis, Sven J. van der Lee, Vincent Chouraki, Johanna Jakobsdottir, Sonia Moreno-Grau, Yuning Chen, Shahzad Ahmad, Merce Boada, Annette L. Fitzpatrick, Albert V. Smith, Xueqiu Jian, Seung-Hoan Choi, Hieab H. Adams, Jennifer A. Brody, Gudny Eiriksdottir, Thor Aspelund, Edith Hofer, Chloe Sarnowski, Charles C. White, Dina Vojinovic, L. Adrienne Cupples, Jayanadra J. Himali, Adela Orellana, Melissa E. Garcia, Honghuang Lin, Hata Comic, Gena Roschupkin, Shuo Li, Isabel Hernández, Qiong Yang, Alexa S. Beiser, Lluis Tarraga, Thomas H. Mosley, WT Longstreth, Jr, Fernando Rivadeneira, Eric Boerwinkle, Jerome I. Rotter, Andre G. Uitterlinden, Itziar de Rojas, Inés Quintela, Ángel Carracedo, Luis M. Real, Yasaman Saba, Oscar L. Lopez, Myriam Fornage, Najaf Amin, Lenore J. Launer, M. Arfan Ikram, Helena Schmidt, Reinhold Schmidt, Anita L. DeStefano, Vilmundur Gudnason, Bruce M. Psaty, Agustin Ruiz, Cornelia M. van Duijn, Sudha Seshadri
