# Supplementary material for: Gene-based analysis in HRC imputed genome wide association data identifies three novel genes for Alzheimer’s disease
Source: PLoS One. 2019 Jul 8;14(7):e0218111. doi: 10.1371/journal.pone.0218111 (PMC6613773; doi:10.1371/journal.pone.0218111)
Supplement: S1 Table — (PDF) [file pone.0218111.s002.pdf]

S1 Table. POLARIS Gene-Based Results for GWAS Associated Genes

Gene	Chr	No. of SNPs	Beta	SE	P-value
CR1	1	288	0.228	0.0827	0.0059
BIN1	2	342	0.322	0.0848	0.0001
INPP5D	2	474	0.005	0.0670	0.9449
MEF2C	5	369	-0.032	0.0672	0.6375
HLA-DRB5	6	9	1.308	0.3495	0.0002
CD2AP	6	414	0.237	0.0654	0.0003
NME8	7	398	0.060	0.0922	0.5140
ZCWPQ1	7	81	0.168	0.2009	0.4031
EPHA1	7	59	0.374	0.1649	0.0235
PTK2B	8	492	0.035	0.0576	0.5446
CLU	8	158	0.461	0.0988	3.0×10^{-6}
CELF1	11	162	0.445	0.1508	0.0031
MS4A6A	11	102	0.288	0.0873	0.0010
PICALM	11	436	0.240	0.0943	0.0109
SORL1	11	347	-0.119	0.0617	0.0540
FERMT2	14	246	0.215	0.1562	0.1680
SLC24A4	14	587	0.266	0.1475	0.0711
RIN3	14	506	0.436	0.1897	0.0216
DSG2	18	273	-0.155	0.1411	0.2726
ABCA7	19	76	0.541	0.1265	1.94×10^{-5}
CD33	19	60	0.095	0.2527	0.7066
CASS4	20	193	0.214	0.1734	0.2165
