# Supplementary material for: Gene-based analysis in HRC imputed genome wide association data identifies three novel genes for Alzheimer’s disease
Source: PLoS One. 2019 Jul 8;14(7):e0218111. doi: 10.1371/journal.pone.0218111 (PMC6613773; doi:10.1371/journal.pone.0218111)
Supplement: S2 Table — (PDF) [file pone.0218111.s003.pdf]

S2 Table. Gene-Wide Significant Genes from POLARIS Gene-based Analysis Conditioned on *APOE* and *BCAM* and best IGAP SNP and p-value

			POLARIS, conditioned on *APOE* and *BCAM*			IGAP	
Chr	Gene	No. of SNPs	Beta	SE	P-value	SNP	P-value
4	*PPARGC1A*	480	0.896	0.1896	2.3×10^{-6}	rs11734408	0.001345
8	*SCARA3 (CLU)*	240	0.549	0.1097	5.6×10^{-7}	rs7982	2.48×10^{-7}
15	*RORA*	1813	0.347	0.0693	5.6×10^{-7}	15-61211520	5.26×10^{-6}
16	*ZNF423*	1056	0.549	0.1194	4.3×10^{-6}	16-49888847	0.0003226
19	*BCL3*	88	0.167	0.0676	0.0133	rs12459810	6.77×10^{-43}
19	*CBLC*	50	0.151	0.1243	0.2237	rs2889414	$2.49 \times 10-43$
19	*BCAM*	71	NA	NA	NA	rs28399637	4.66×10^{-69}
19	*PVRL2*	160	0.255	0.0644	7.4×10^{-5}	rs6857	2.50×10^{-575}
19	*TOMM40*	108	0.335	0.0897	0.0002	rs6857	2.50×10^{-575}
19	*APOE*	55	NA	NA	NA	rs6857	2.50×10^{-575}
19	*APOC1*	34	-0.146	0.1045	0.1621	rs6857	2.50×10^{-575}
19	*APOC4-APOC2*	62	0.435	0.0897	1.2×10^{-6}	rs429358	6.70×10^{-536}
