# Supplementary material for: Gene-based analysis in HRC imputed genome wide association data identifies three novel genes for Alzheimer’s disease
Source: PLoS One. 2019 Jul 8;14(7):e0218111. doi: 10.1371/journal.pone.0218111 (PMC6613773; doi:10.1371/journal.pone.0218111)
Supplement: S3 Table — (PDF) [file pone.0218111.s004.pdf]

S3 Table. GERAD Cohort Descriptives and Sample Size

	**TOTAL**	MRC	ART	BONN	WASHU	NIMN	UCL-Laser	UCL-PRION	HNR	1958C	NBS	KORA
**AD Cases**												
n_{total}	3332	1009	960	555	423	127	47	211	0	0	0	0
% Female	63.7	70.4	60.9	63.8	56	63	74.5	58.3	-	-	-	-
Mean Age	77.9	80.9	76.6	72.9	82.1	80.1	80.6	63.6	-	-	-	-
**Controls**												
n_{total}	9832	873	82	37	233	0	0	0	353	5343	2477	434
% Female	51.6	61.6	59.8	64.9	66.5	-	-	-	53	49.8	50.5	49.1
Mean Age	51.1	75.9	77.9	79.5	78.5	-	-	-	54.6	45	-	56
Total by cohort	13164	1882	1042	592	656	127	47	211	353	5343	2477	434
